# Relative HPV Viral Load Measured by ΔCt for Risk Stratification in Cervical Cancer Screening

**DOI:** 10.3390/cancers18162568

**Published:** 2026-08-10

**Authors:** Morena d’Avenia, Laila Sara Arroyo Mühr, Marcella Mastromauro, Federica Spadaccino, Elisabetta Razzuoli, Michela Iacobellis, Filippo Dell’Anno

**Affiliations:** 1 UOSVD Cytopathology and Screening, ASL BARI, Di Venere Hospital, 70131 Bari, Italy; marcella.mastromauro@asl.bari.it (M.M.); federica.spadaccino@asl.bari.it (F.S.); michela.iacobellis@asl.bari.it (M.I.); 2 Department of Medicine and Surgery, University of Milano-Bicocca, 20900 Monza, Italy; 3 Center for Cervical Cancer Elimination, Department of Clinical Science, Intervention and Technology (CLINTEC), Karolinska Institutet, 14186 Stockholm, Sweden; sara.arroyo.muhr@ki.se; 4 Centro di Referenza di Oncologia Veterinaria e Comparata (CEROVEC), Istituto Zooprofilattico Sperimentale Piemonte Liguria Valle D’Aosta (IZSPLV), 16129 Genoa, Italy; filippo.dellanno@izsplv.it

**Keywords:** human papillomavirus (HPV), relative viral load, cervical cancer screening, cervical intraepithelial neoplasia, risk stratification

## Abstract

**Background/Objectives:** Human papillomavirus (HPV) testing is widely used in cervical cancer screening because of its high sensitivity, although its limited specificity requires effective triage strategies. This study aimed to evaluate the association between relative viral load (RVL) and cervical lesion severity, focusing on HPV-positive women with non-high-grade cytology. **Methods:** The study included 997 women with histological outcomes from a cohort of 2765 HPV-positive individuals identified among 37,030 women screened in the Bari metropolitan area (Italy) using a real-time HPV-DNA test. Associations between RVL, estimated using the ΔCt method, and lesion severity were assessed using histological and cytological classifications. Logistic regression models evaluated the independent effect of RVL, adjusting for age and HPV detection channel. **Results:** Lower ΔCt values (indicating higher RVL) were significantly associated with high-grade lesions (CIN2+) (OR = 0.91, 95% CI: 0.89–0.94, *p* < 0.001). The association was observed for HPV16 and the pooled 12-high-risk HPV detection channel, while no significant association was observed for HPV18. A similar association was observed when cytological classification was used as the outcome. Age showed a modest effect, with reduced odds of high-grade lesions observed in women aged ≥50 years. Among women with non-high-grade cytology who subsequently underwent histological assessment, 17.6% had underlying CIN2+, and RVL remained significantly associated with lesion severity in this subgroup. However, ΔCt showed only modest discrimination for high-grade lesions [AUC = 0.643 (95% CI: 0.605–0.681)]. **Conclusions:** Higher HPV RVL is associated with increased lesion severity, particularly for HPV16 and the pooled 12-high-risk detection channel, but not for HPV18. Given its modest standalone discriminatory performance and the selected nature of the histologically verified cohort, ΔCt should be considered an exploratory marker rather than a validated triage tool. Prospective validation is required to determine whether it provides incremental clinical value.

## Simple Summary

Human papillomavirus (HPV) infection is the primary cause of cervical cancer, but most HPV-positive women do not develop clinically significant lesions. Identifying those at higher risk is therefore essential to improve the management of HPV-positive screening results. In this study, we investigated whether the amount of HPV DNA in cervical samples, normalized to cellular content using the routine real-time PCR parameter ΔCt, was associated with the severity of cervical lesions. Lower ΔCt values, indicating a higher relative HPV DNA burden, were associated with high-grade cervical lesions, particularly for HPV16 and the pooled 12-high-risk HPV detection channel, but not for HPV18. A similar association was observed among women with non-high-grade triage cytology who subsequently underwent histological assessment. However, ΔCt showed only modest ability to distinguish between women with and without high-grade lesions. These findings support further investigation of ΔCt as an exploratory, low-cost marker that can be derived from routine HPV testing, although prospective studies are required before it can be incorporated into cervical cancer screening algorithms.

## 1. Introduction

Human papillomavirus (HPV) DNA-based testing is used worldwide for primary cervical cancer screening. It is recommended by WHO and European guidelines in women aged 30–64 years [1,2], due to its high sensitivity in detecting cervical intraepithelial neoplasia grade 2 or worse (CIN2+) and cervical cancer, as well as its suitability for high-throughput screening. Large, randomized trials and meta-analyses have demonstrated that HPV testing provides superior long-term protection against cervical cancer compared with cytology-based screening alone [3,4]. However, the sensitivity of HPV testing is accompanied by reduced specificity, resulting in a substantial proportion of HPV-positive women who will not develop clinically significant disease. Consequently, effective triage strategies are essential to identify those women at highest risk who warrant colposcopic follow-up.

Reflex cytology remains the most widely implemented triage tool in HPV-based screening algorithms, either at baseline or during follow-up. Nonetheless, cytology is subjective, requires experienced personnel, and shows limited sensitivity [2,5]. For these reasons, considerable effort has been devoted to identifying alternative or complementary biomarkers for risk stratification, including HPV genotyping, p16/Ki-67 dual staining, host and viral DNA methylation, and HPV viral load [6,7,8,9].

Among these candidates, HPV viral load has long attracted interest as a potential indicator of lesion severity. Several studies have reported that higher HPV viral load is associated with high-grade cytological abnormalities and CIN2+ lesions, suggesting that viral burden may reflect the number of infected cells and the extent of viral replication and may be associated with the subsequent risk of high-grade lesions or vertical transmission [10,11,12,13,14].

However, other investigations have failed to demonstrate a consistent or clinically useful association, particularly when viral load is evaluated in cross-sectional screening settings rather than longitudinal cohorts, and associations may differ across HPV genotypes [15,16,17]. These conflicting findings have raised questions about the robustness and clinical applicability of viral load as a triage biomarker.

Heterogeneity in results may be due to the lack of methodological standardization in viral load assessment. In some studies, viral load has been calculated as HPV copies per mL of transport medium or inferred directly from the cycle threshold (Ct) value of the HPV target in real-time PCR assays, assuming that lower Ct values correspond to a higher viral burden [10,12]. While analytically straightforward, this approach does not account for variability in sample cellularity, DNA input, or sampling efficiency, all of which can substantially influence Ct values and the reliability of target detection [18]. Other studies have attempted to overcome these limitations by normalizing HPV signals to specimen volume, cell counts, or cellular housekeeping genes, providing a relative measure of viral load that better reflects HPV DNA content in relation to the number of collected cells [15,19,20].

Therefore, viral-load estimates remain relative and are influenced by sample composition, although these effects may be only partially corrected by normalization approaches such as ΔCt.

The present study aimed to evaluate the association between HPV relative viral load (RVL), estimated using the ΔCt method and normalized to a cellular internal control, and the severity of cytological and histological cervical lesions in a large retrospective screening cohort. Furthermore, we explored whether the association between RVL and lesion severity was also present among HPV-positive women with non-high-grade cytology who subsequently underwent histological assessment.

## 2. Materials and Methods

### 2.1. Study Population and Sample Collection

The study population was drawn from the population-based Regional Cervical Cancer Screening Program of the Apulia Region, Italy. This study analyzed 37,030 women who underwent cervical cancer screening between January and December 2023 in the metropolitan area of Bari. Eligible women, aged 30–64 years, participated in HPV-based primary cervical cancer screening. Women with a history of total hysterectomy, pregnancy, immunosuppression, or HIV infection were not eligible for routine primary HPV screening under the Regional Cervical Cancer Screening Program and were therefore not included in the study population. In the present study, women with a history of treatment for CIN2+ were excluded. HPV vaccination status was not used as an exclusion criterion because the routinely vaccinated birth cohorts had not yet reached the age range eligible for primary HPV-based screening during the 2023 study period.

Following the manufacturer’s standard guidelines, cervical specimens were obtained by trained midwives at 45 public screening clinics using Cervex-Brush^®^ (Rovers Medical Devices, Oss, The Netherlands) and PreservCyt (Hologic Inc., Marlborough, MA, USA) vials (20 mL). Testing was centralized in a single laboratory responsible for conducting both the HPV DNA molecular analysis and cytological triage.

HPV-positive women with positive triage cytology, defined as atypical squamous cells of undetermined significance or worse (≥ASC-US), were referred to colposcopy within 1–6 months after the baseline screening result.

When colposcopy showed grade 1 or higher findings, cervical biopsies were usually performed, and women diagnosed with CIN2+ [21] subsequently underwent surgical treatment by conization. HPV-positive women with negative triage cytology underwent repeat HPV testing after one year. If HPV positivity persisted, women were referred to colposcopy and, when indicated, to biopsy and subsequently to conization. For all analyses, the ΔCt value was derived from the baseline HPV screening specimen.

Collected data included HPV test results, including Ct values for the HPV targets and the β-globin internal control used to estimate the relative HPV DNA burden, liquid-based cytological results, histological findings from biopsy or cone specimens, when available, and age.

### 2.2. HPV-DNA Assay

Samples were analyzed using the qualitative Cobas^®^ 4800 HPV Test (Roche Molecular Systems Inc., Pleasanton, CA, USA), which individually detects HPV16 and HPV18 while reporting 12 additional high-risk HPV types as a pooled detection channel. The assay amplifies the human β-globin gene as an internal control to confirm the presence of human nucleated cells and assess potential assay inhibition.

After vortexing and decapping, the primary collection tubes were directly sampled by the Cobas x480 fluid handler. The extraction workflow processed 400 μL of specimen to generate a 150 μL DNA eluate, of which 25 μL were used for PCR amplification.

Cycle threshold (Ct) values for HPV16, HPV18, and the pooled 12-high-risk HPV channel were reported by the software. Clinical cut-off values were set at Ct < 40.5 for HPV16, Ct < 40 for HPV18, and Ct < 40 for the pooled 12-high-risk HPV channel, in accordance with published data [22], to maintain the validated clinical performance of the assay. The manufacturer’s cut-off value for the β-globin internal control was Ct = 40.

HPV RVL was estimated from real-time PCR by calculating the ΔCt value (Ct HPV − Ct β-globin), with lower ΔCt values indicating a higher relative HPV DNA burden. RVL was analyzed as a continuous variable.

Because the Cobas^®^ 4800 HPV Test is a qualitative clinical assay, it is not validated by the manufacturer for quantitative viral-load measurement. However, previous studies have used Cobas 4800 Ct values as a semiquantitative proxy for HPV viral burden [23,24]. Channel-specific amplification efficiencies were not evaluated in the present study; therefore, ΔCt was interpreted as an exploratory semiquantitative proxy for relative HPV DNA burden.

For participant-level analyses, each woman contributed a single ΔCt value. In women with positivity in multiple channels, we applied a hierarchical assignment based on a prespecified clinical risk hierarchy of the Cobas detection channels: HPV16, followed by HPV18, and then the pooled 12-high-risk HPV channel. Thus, the ΔCt corresponding to the highest-priority positive channel was used in the overall analysis [25].

### 2.3. Liquid-Based Cytology

Liquid-based cytology (LBC) was performed using the ThinPrep^®^ 2000 Processor (Hologic Inc., Marlborough, MA, USA), following the manufacturer’s instructions. Cytological interpretations adhered to the Bethesda System with Negative for Intraepithelial Lesions or Malignancy (NILM) classified as a negative result. Specimens with low cellularity (<5000 cells per slide), obscuring blood, mucus, or inflammatory exudate, or amorphous material were classified as inadequate or non-diagnostic. All remaining categories—ASC-US, atypical glandular cells (AGC), low-grade squamous intraepithelial lesion (L-SIL), atypical squamous cells, cannot exclude HSIL (ASC-H), high-grade squamous intraepithelial lesion (H-SIL), squamous cell carcinoma (SCC), and adenocarcinoma (AC)—were considered cytologically abnormal.

For statistical analyses, cytological results were grouped into two categories: non-high-grade cytology, including NILM, ASC-US, and L-SIL; and high-grade cytology, including AGC, ASC-H, H-SIL, SCC, and AC. AGC cases were assigned to the high-grade category [21]. Although AGC does not invariably correspond to high-grade histology, a sensitivity analysis excluding AGC from the high-grade cytology category [26,27] was also performed.

This grouping was defined to reflect risk stratification rather than strict diagnostic classification. As part of the local HPV-based primary screening algorithm, LBC was used for triage and follow-up of HPV-positive women.

### 2.4. Histology

Cervical biopsies were obtained from the most suspicious areas during colposcopy. Women diagnosed with CIN2+ on biopsy underwent conization, when clinically indicated, using either cold knife conization or loop electrosurgical excision procedure.

Biopsy and cone specimens were formalin-fixed, paraffin-embedded, sectioned, and stained with hematoxylin and eosin following standard histopathological procedures. Histological slides were independently reviewed by two expert pathologists in a centralized laboratory. Lesions were classified according to the WHO 2020 Classification of Tumors. Inflammatory and reactive changes were considered negative for the purposes of this analysis. For statistical analyses, histological lesions were grouped into non–high-grade (≤CIN1, including koilocytic atypia and non-neoplastic findings) and high-grade (CIN2+, including CIN2, CIN3, SCC and AC) [21].

### 2.5. Statistical Analysis

Descriptive statistics, including medians, interquartile ranges, and frequencies, were calculated for ΔCt values according to lesion grade and HPV detection channel. Differences in ΔCt distribution between lesion grade groups were assessed using the Wilcoxon rank-sum test because ΔCt values were not normally distributed.

ROC curve analysis was performed to evaluate the ability of ΔCt to discriminate between non-high-grade and high-grade lesions. The area under the ROC curve was reported with its 95% confidence interval.

Analyses were conducted separately for histological and cytological outcomes. The association between ΔCt and lesion severity was assessed using logistic regression models, with ΔCt included as a continuous predictor. Both unadjusted models and models adjusted for age category (30–39, 40–49, and 50–64 years) were fitted. These decade-based age strata were selected a priori to provide clinically interpretable groups while maintaining adequate numbers of women and histological outcomes within each stratum. This categorization is also consistent with previously reported age-related differences in HPV positivity rates within Italian screening populations [18,28].

Logistic regression analyses were performed using complete-case data. Overall, only two observations had missing ΔCt values and were therefore excluded from the relevant regression analyses. The number of observations included in each model is reported.

To explore detection-channel-specific effects, separate multivariable logistic regression models were fitted among women positive for HPV16, HPV18, and the pooled 12-high-risk HPV channel. Each model included ΔCt as a continuous predictor and was adjusted for age category. The assumption of linearity between continuous ΔCt and the log-odds of high-grade lesions was assessed using restricted cubic splines. As the spline model did not significantly improve model fit compared with the linear model, ΔCt was retained as a continuous linear predictor in all logistic regression analyses. Detection-channel-specific analyses were prespecified based on the predefined classification of HPV genotypes provided by the assay (HPV16, HPV18, and pooled 12-high-risk HPV types), reflecting the primary biological and clinical stratification of interest.

All analyses were performed using Stata version 17 (StataCorp, College Station, TX, USA). A two-sided *p*-value < 0.05 was considered statistically significant.

### 2.6. Ethics Approval and Consent to Participate

The study received ethical approval from the Local Ethics Committee (Ref. 7438/CEL/2023, addendum 1809/CEL/2024) and was conducted in accordance with national screening regulations. Participants signed an informed consent form authorizing the use of their clinical and epidemiological data in anonymized and aggregated form for scientific research purposes, in compliance with the General Data Protection Regulation (GDPR).

## 3. Results

### 3.1. Study Cohort Characteristics

A total of 37,030 women were screened for cervical cancer (median age 48, IQR: 30–64), and 2765/37,030 (7.47%) tested HPV-positive (median age 44, IQR: 30–64). The distribution of HPV detection channels showed 334 women positive for HPV16 only, 87 for HPV18 only, and 2196 for the pooled 12-high-risk HPV channel. Co-infections included HPV16/18 (*n* = 5), HPV16/pooled 12-high-risk HPV channel (*n* = 105), HPV18/pooled 12-high-risk HPV channel (*n* = 34), and triple positivity (*n* = 4).

**Figure 1:**
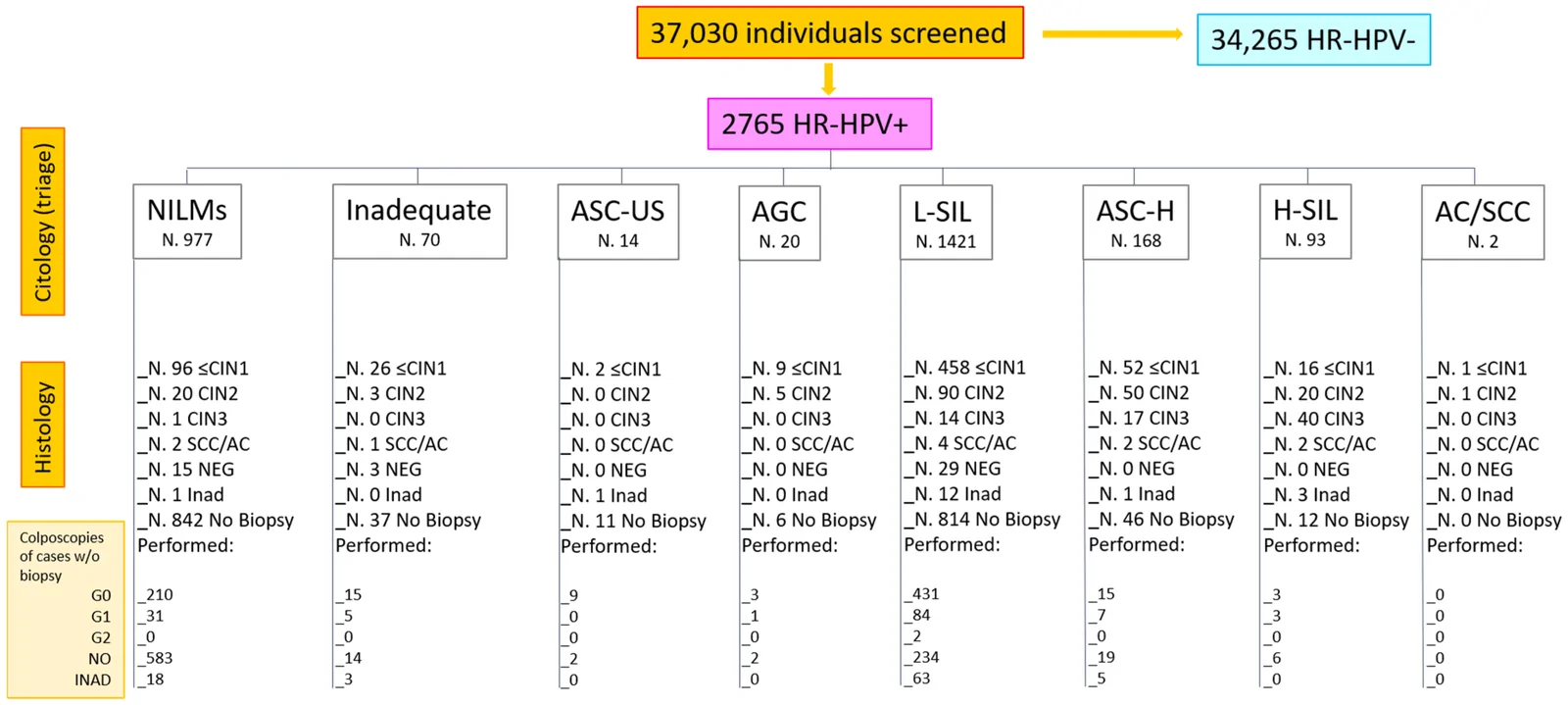
Distribution of liquid-based cytology results and corresponding histological diagnoses among HPV-positive women included in the study. Histological outcomes are stratified as non-high-grade (≤CIN1), high-grade (CIN2, CIN3), Squamous Cell Carcinoma (SCC), Adenocarcinoma (AC), negative findings (NEG), inadequate samples (Inad), and cases without available histology because of negative colposcopy or missed follow-up. G0: negative colposcopy; G1: grade 1 colposcopic findings; G2: grade 2 colposcopic findings; NO: did not attend follow-up or colposcopy; INAD: inadequate colposcopy.

Regarding triage, 4.83% (*n* = 1788) had abnormal (≥ASC-US) or inadequate LBC triage and were referred for immediate colposcopy, while 2.63% (*n* = 977) had NILM triage cytology and were scheduled for repeat HPV testing after one year. Among these, at 1-year follow-up, 326 were negative and 443 remained HPV-positive and were referred for colposcopy. Of the 443 women with persistent HPV positivity at one year, 394 attended follow-up investigations and 135 underwent their first biopsy after a median interval of 1.46 years (IQR: 1.30–1.97) following their baseline HPV test. The remaining HPV-positive women with NILM cytology (*n* = 208) missed their 1-year-follow-up test. Overall, 860 women across all screening pathways did not complete further follow-up. Figure 1 summarizes HPV status, triage cytology, histological outcomes, and colposcopic findings among women who did not undergo histological assessment.

Among women with available histological outcomes (*n* = 997), histology identified 707 (70.9%) non-high-grade lesions (CIN 1, koilocytic atypia, negative or inflammatory findings, polyps, and benign or reactive squamous abnormalities, including metaplasia) and 272 (27.3%) high-grade lesions (CIN2, CIN3, AC and SCC); 18 results were inadequate. The median ΔCt was 2.8 (IQR: −1.4 to 7.3), ranging from −11.9 to 14.3. Among women with available histology, baseline triage cytology was non-high-grade in 745 women (74.7%; NILM, ASC-US, or L-SIL), high-grade in 219 women (22.0%; AGC, ASC-H, H-SIL, SCC, or adenocarcinoma), and inadequate in 33 women (3.3%). Single-channel positivity was observed in 933 (93.6%) women and multiple channel positivity in 64 (6.4%).

Baseline characteristics of HPV-positive women with and without histological verification are reported in Supplementary Table S1. As expected, women undergoing histological assessment showed markedly different cytological distributions and lower ΔCt values [ΔCt, median (IQR) 2.8 (−1.4–7.3) vs. 4.9 (0.3–9.0), *p* < 0.001], whereas age distributions were comparable between groups (*p* = 0.085).

The distributions of HPV detection-channel positivity are also presented in Supplementary Table S1; channel counts include women with multiple-channel positivity.

The distribution of histological diagnoses according to the number of positive HPV detection channels is summarized in Table 1.

### 3.2. Relative Viral Load Correlates with Lesion Grade but Exhibits Modest Discriminative Performance

The association between HPV detection channel and RVL, expressed as ΔCt values, was evaluated according to histological lesion grade. Lower ΔCt values (indicating higher RVL) were observed in high-grade compared with non-high-grade lesions, with detection channel-specific differences.

For HPV16, median ΔCt values were significantly lower in high-grade than in non-high-grade lesions (0.4 [IQR: −2.7 to 3.7] vs. 4.85 [IQR: 0.3 to 8.6], *p* = 0.0001). A similar pattern was observed for the pooled 12-high-risk HPV channel (0.6 [IQR: −3.0 to 4.75] vs. 3.6 [IQR: −0.5 to 8.1], *p* = 0.0001). In contrast, no significant difference was observed for HPV18 (0.05 [IQR: −3.65 to 7.2] vs. 4.2 [IQR: 0.9 to 8.1], *p* = 0.1925) (Table 2).

A similar association was observed when cytological classification was used as the outcome (Supplementary Table S2). Wilcoxon rank-sum tests confirmed significantly lower median ΔCt values in high-grade than in non-high-grade lesions for both histological outcomes (1.08 vs. 3.69; z = 6.917; *p* < 0.001) and cytological outcomes (1.09 vs. 3.38; z = 5.54; *p* < 0.001). Receiver operating characteristic (ROC) curve analysis demonstrated that ΔCt had only modest ability to discriminate between non-high-grade and high-grade cervical lesions, yielding an area under the curve (AUC) of 0.643 (95% CI: 0.605–0.681). To further characterize the diagnostic performance of ΔCt, an additional threshold analysis was performed based on the ROC curve. The threshold corresponding to the maximum Youden index was ΔCt = 1.0. At this threshold, the sensitivity was 55.9%, specificity 67.6%, positive predictive value (PPV) 39.8%, and negative predictive value (NPV) 80.0% (Supplementary Table S3).

### 3.3. Lower ΔCt Values Are Associated with Increased Odds of High-Grade Lesions Independently of Age

Logistic regression analyses showed that lower ΔCt values were associated with higher odds of high-grade lesions. In the unadjusted model, each unit increase in ΔCt was associated with an 8% reduction in the odds of high-grade lesions (OR = 0.92, 95% CI 0.89–0.94, *p* < 0.001).

Women aged 50–64 years had lower odds of high-grade lesions than women aged 30–39 years (OR = 0.51, 95% CI 0.36–0.74, *p* < 0.001), whereas no significant difference was observed for women aged 40–49 years (Table 3).

**Figure 2:**
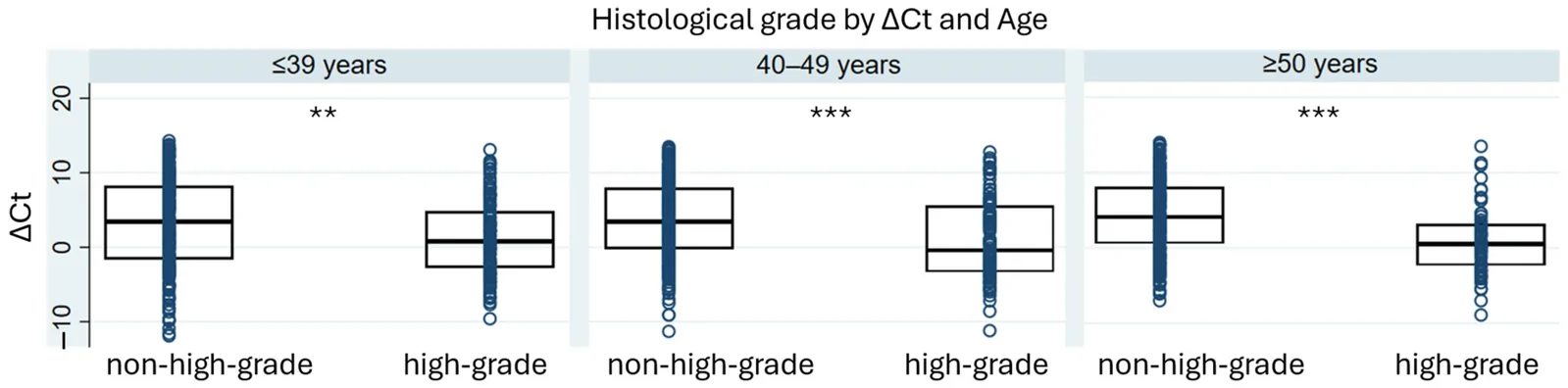
Distribution of ΔCt values according to histological lesion grade within each age category. High-grade lesions (CIN2+) showed lower ΔCt values across all age categories. Comparisons were performed using the Wilcoxon rank-sum test (** *p* < 0.01; *** *p* < 0.001).

In the adjusted model, ΔCt remained significantly associated with lesion severity (OR = 0.91, 95% CI 0.89–0.94, *p* < 0.001). Women aged 50–64 years continued to have lower odds of high-grade lesions than women aged 30–39 years (OR = 0.55, 95% CI 0.38–0.79, *p* = 0.001). Similar results were observed when cytological classification was used as the outcome (Supplementary Table S4). ΔCt values were consistently lower in high-grade lesions across all age categories, in agreement with the inverse association observed in logistic regression models (Figure 2).

### 3.4. Detection-Channel Stratification Shows That Lower ΔCt Is Associated with High-Grade Lesions for HPV16 and the Pooled 12-High-Risk HPV Channel, but Not for HPV18

**Figure 3:**
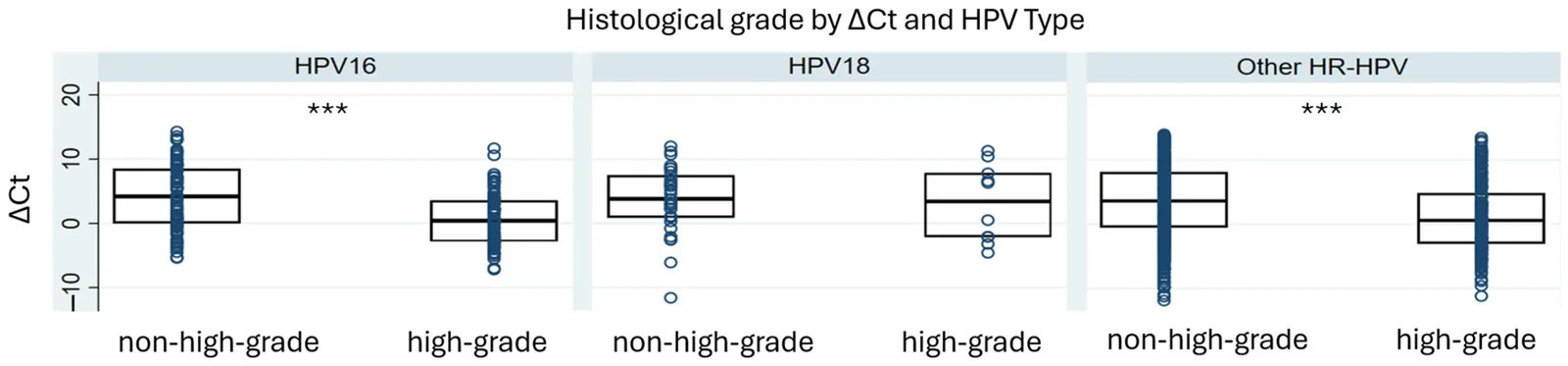
Detection-channel-stratified distribution of ΔCt values according to histological lesion grade. Box-and-strip plots show ΔCt distributions in non-high-grade (≤CIN1) and high-grade lesions (CIN2+) for HPV16, HPV18, and the pooled 12-high-risk HPV detection channel. Comparisons were performed using Wilcoxon rank-sum tests. ΔCt values were significantly lower in high-grade lesions for HPV16 and the pooled 12-high-risk HPV channel (*** *p* < 0.001), whereas no significant difference was detected for HPV18.

Detection-channel-specific logistic regression analyses were performed separately among women positive for HPV16, HPV18, or the pooled 12-high-risk HPV types and adjusted for age category (Table 4). Lower ΔCt values were significantly associated with increased odds of high-grade histological lesions among HPV16-positive women (OR = 0.87, 95% CI 0.82–0.92, *p* < 0.001) and among women positive for the pooled 12-high-risk HPV types (OR = 0.93, 95% CI 0.90–0.95, *p* < 0.001). In contrast, no significant association was observed among HPV18-positive women (OR = 0.93, 95% CI 0.84–1.03, *p* = 0.167). These findings were consistent with the detection-channel-specific comparisons of ΔCt distributions presented in Table 2 and Figure 3.

Univariate comparisons of ΔCt distributions between histological grades within each HPV detection channel showed significantly lower ΔCt values in high-grade lesions for HPV16 and the pooled 12-high-risk HPV channel, whereas no significant difference was observed for HPV18 (Figure 3).

### 3.5. Association Between Lower ΔCt and Histological Severity Among Women with Non-High-Grade Cytology

Among 745 women with non-high-grade cytology who subsequently underwent histological assessment, 131 cases (17.6%) had high-grade histological lesions.

ΔCt values differed significantly between histological groups, with lower median ΔCt observed in women with high-grade histological lesions compared with those with non-high-grade lesions (0.95 [IQR: −2.3 to 6.15] vs. 3.7 [IQR: −0.2 to 8.2], *p* = 0.0001).

**Figure 4:**
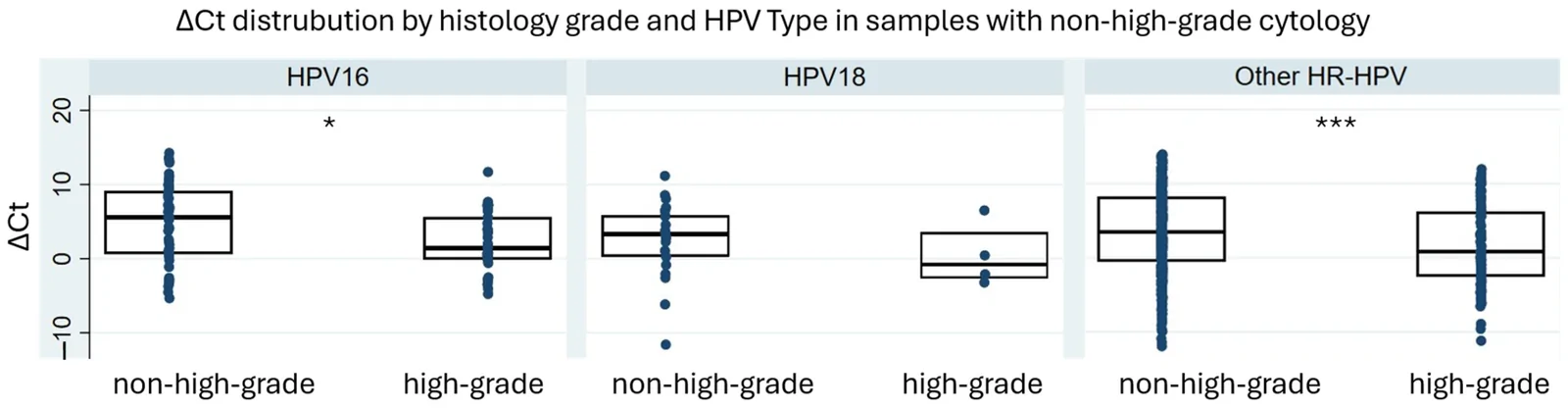
Association between HPV relative viral load, estimated using ΔCt, and histological severity in women with non-high-grade cytology who subsequently underwent histological assessment. Box-and-strip plots show ΔCt distributions according to histological outcome—non-high-grade ≤CIN1) or high-grade (CIN2+)—overall and for HPV16, HPV18, and the pool 12-high-risk HPV detection channel. ΔCt values differed significantly between histological groups for HPV16 (* *p* < 0.05) and the pooled 12-high-risk HPV channel (*** *p* < 0.001), whereas no significant association was observed for HPV18.

Detection-channel-specific analyses showed heterogeneous patterns. ΔCt distributions differed significantly between histological groups for HPV16 (*p* = 0.017) and the pooled 12-high-risk HPV channel (*p* = 0.001), whereas no significant difference was observed for HPV18 (*p* = 0.331; Figure 4).

Logistic regression analysis showed that higher ΔCt values were associated with lower odds of high-grade histology in the overall subgroup (OR = 0.94; 95% CI 0.90–0.97; *p* < 0.001). Detection-channel-specific analyses showed corresponding associations among HPV16-positive women (OR = 0.89, 95% CI 0.83–0.97; *p* = 0.005) and women positive for the pooled 12-high-risk HPV channel (OR = 0.94, 95% CI 0.90–0.98; *p* = 0.001). No significant association was observed amongHPV18-positive women (OR = 0.94, 95% CI 0.82–1.08; *p* = 0.419).

## 4. Discussion

In this large screening cohort, relative viral load (RVL), estimated using ΔCt values, was consistently associated with lesion severity across both histological and cytological classifications, with clear detection-channel-specific differences. Lower ΔCt values (reflecting higher RVL) were associated with high-grade lesions, particularly for HPV16 and the pooled 12-high-risk HPV detection channel. Although this latter group includes different genotypes with varying oncogenic potential (including those in IARC groups 1c and 1d [29]), the association between ΔCt and lesion severity remained statistically significant.

These findings are consistent with previous studies reporting a positive association between HPV viral load and lesion severity, especially for HPV16 [30,31,32]. The observed heterogeneity across genotypes is also in line with prior evidence, suggesting differences in viral biology and integration patterns, particularly for HPV18 [33,34]. In this context, the lack of a significant association for HPV18 may reflect both limited statistical power and detection-channel-specific biological features.

Beyond sample size constraints, HPV18 undergoes viral integration into the host genome significantly earlier and more frequently than HPV16. This integration often disrupts or deletes parts of the viral genome, markedly reducing detectable viral DNA copies despite active oncogenic expression. Consequently, HPV18 DNA levels in exfoliated cells do not necessarily correlate with lesion severity.

Additionally, HPV18 exhibits a distinct tropism for glandular cells and is frequently associated with endocervical lesions developing deeper within the cervical canal. These lesions are often less efficiently collected by routine ectocervical sampling, leading to highly variable viral load measurements. Prior literature confirms this weak, non-linear relationship between HPV18 viral load and lesion severity [35,36,37]. Overall, our findings do not support the use of ΔCt for HPV18-specific risk stratification.

An exploratory finding of this study was that the association between ΔCt and histological severity was also observed among women with non-high-grade cytology who subsequently underwent histological assessment. However, because this subgroup was clinically selected and the standalone discrimination of ΔCt was modest, these findings should not be interpreted as evidence that ΔCt can guide colposcopy referral.

The modest discriminative performance of ΔCt likely reflects, at least in part, the influence of sample composition. While low cellularity primarily affects the likelihood of detecting HPV below the assay’s limit of detection [18], it also impacts the quantitative interpretation of Ct values. Since ΔCt represents HPV DNA relative to total cellular DNA [15,18,19,20], the use of a non-specific normalization target does not account for differences in cellular composition, because DNA-based normalization does not distinguish between epithelial and non-epithelial cells. In particular, the presence of inflammatory cells or blood contamination may increase the β-globin signal, leading to an underestimation of relative viral load. Accordingly, ΔCt should be interpreted as an exploratory proxy for relative HPV DNA burden rather than as a direct measure of viral load.

High-grade lesions were observed across a broad range of ΔCt values, further illustrating the limitations of relying on a single quantitative threshold. These findings underscore the need for improved normalization strategies that account for sample cellularity and biological composition. Because ΔCt can be derived from routinely generated real-time PCR data without additional laboratory procedures, it may warrant further investigation in settings where access to more complex triage methods is limited. Nevertheless, further work is needed to assess reproducibility across different operators and sampling conditions, as inter-sample variability remains an important challenge for potential clinical application.

The retrospective design and heterogeneous interval between baseline HPV testing and histological assessment preclude conclusions regarding the predictive value of ΔCt for lesion progression. In addition, ΔCt does not capture other relevant aspects of HPV biology, such as viral transcriptional activity or integration status, which may also influence disease progression.

## 5. Limitations

Several limitations should be considered when interpreting our findings.

*Differential verification bias.* Histological outcomes were not systematically obtained for all HPV-positive women but depended on the routine screening pathway. Women with abnormal cytology underwent immediate colposcopy, whereas those with NILM cytology underwent histological verification only after documented HPV persistence at one year, colposcopic evaluation, and a clinical indication for biopsy. Consequently, the histologically verified subgroup was highly selected and enriched for women at greater baseline risk of cervical disease. This selection is reflected in the lower ΔCt values and slightly higher β-globin Ct values observed among women with available histology compared with those without histology (Supplementary Table S1). Therefore, the observed CIN2+ frequency and the estimated associations cannot be generalized to the full HPV-positive screening population.

*Use of ΔCt as an exploratory semiquantitative proxy.* The Cobas 4800 HPV Test is a qualitative assay and is not validated by the manufacturer for quantitative viral-load measurement. In addition, channel-specific amplification efficiencies were not experimentally evaluated in this study. Although previous studies support an inverse correlation between Cobas Ct values and quantitative HPV burden, ΔCt should be interpreted strictly as an exploratory semiquantitative proxy for relative HPV DNA burden.

*Modest discriminative performance.* The standalone discriminative ability of baseline ΔCt was weak to modest, with an overall AUC of 0.64. Because no clinical threshold was prespecified, and no prospective validation was performed, we did not propose a definitive clinical cutoff. Sensitivity, specificity, and positive and negative predictive values derived from this clinically selected cohort would have limited generalizability. Accordingly, our findings support an association with lesion severity rather than establishing validated clinical triage utility. Demonstration of incremental clinical value would require systematic histological verification and formal comparison with existing triage models, including calibration and decision-curve or net-benefit analyses.

*Heterogeneity in the screening timeline*. The interval between the baseline HPV sample and histological confirmation varied considerably according to the triage pathway, ranging from 1 to 6 months among women with abnormal cytology to more than one year among women with persistent HPV positivity and baseline NILM cytology. The analysis therefore combined relatively short-term and longer-term histological ascertainment, introducing clinical heterogeneity and precluding interpretation as a purely cross-sectional association.

*Handling of multiple-channel positivity and pooled HPV detection.* For women with multiple-channel positivity, a single ΔCt value was assigned using a prespecified hierarchical ranking: HPV16, followed by HPV18, and then the pooled 12-high-risk HPV detection channel. Although this maintained one observation per participant, it simplified the biological complexity of co-infections and did not capture the concurrent viral burden of all positive channels. Potential multiplex PCR channel competition was not experimentally assessed, and a sensitivity analysis restricted to single-channel-positive samples was not performed. In addition, the assay does not distinguish the individual HPV genotypes included in the pooled 12-high-risk HPV detection channel, preventing genotype-specific conclusions within this group.

*Residual confounding and unavailable vaccination status.* Although women ineligible for routine screening, including those who were pregnant, immunosuppressed, HIV-positive, or previously treated for CIN2+, were excluded, individual HPV vaccination status was unavailable in the retrospective screening dataset. Routine vaccination was expected to have had limited influence because fully vaccinated birth cohorts had not yet reached the age eligible for primary HPV screening in 2023; nevertheless, residual confounding cannot be excluded.

*Model assumptions and exploratory subgroup analyses.* ΔCt was modeled as a continuous variable. Potential non-linearity was assessed using restricted cubic splines, and the spline model did not significantly improve model fit compared with the linear model. Analyses within cytological subgroups should nevertheless be considered exploratory because of the clinically selected nature and limited size of these subgroups.

## 6. Conclusions

In conclusion, a higher HPV relative viral load, as estimated using ΔCt, was associated with greater cervical lesion severity for HPV16 and the pooled 12-high-risk HPV detection channel, but not for HPV18.

Given its modest standalone discriminative performance and the selected nature of the histologically verified cohort, ΔCt should be considered an exploratory marker rather than a validated triage tool. Prospective studies with systematic histological verification and standardized analytical methods are required to determine whether ΔCt provides incremental value beyond established triage approaches.

## Figures and Tables

**Table 1:** Distribution of histological diagnoses according to HPV target detection (single, double, and triple positivity).

Histological Diagnosis	Single Target (*n*)	Double Target (*n*)	Triple Target (*n*)	Total (*n*)
CIN1	378	23	1	402
CIN2	171	16	2	189
CIN3	64	7	1	72
SCC/AC	10	1	0	11
Koilocytic atypia	181	9	0	190
Negative histology	52	1	0	53
Polyp	29	0	0	29
Inflammatory changes	18	1	0	19
Squamous abnormalities	14	0	0	14
Inadequate	16	2	0	18

**Table 2:** Association between HPV genotype, histological lesion grade, and relative viral load (RVL), expressed as ΔCt values (median (IQR)). Lower ΔCt values indicate higher viral load.

HPV Type	Histological Lesion Grade	*n*. of Subject	Median ΔCt (IQR)	*p*-Value
HPV16	Non-high-grade	102	4.85 (0.3–8.6)	R
	High-grade	98	0.40 (−2.7–3.7)	0.0001
HPV18	Non-high-grade	42	4.2 (0.9–8.1)	R
	High-grade	16	0.05 (−3.65–7.2)	0.1925
Pooled 12-HR-HPV channel	Non-high-grade	600	3.6 (−0.5–8.1)	R
	High-grade	188	0.6 (−3.0–4.75)	0.0001

**Table 3:** Logistic regression analysis of the association between ΔCt and histological lesion grade, with and without adjustment for age category.

Predictor	Model	OR (95% CI)	*p*-Value
ΔCt	Unadjusted	0.92 (0.89–0.94)	<0.001
Age 40–49 years	Age only	0.81 (0.59–1.12)	0.208
Age 50–64 years	Age only	0.51 (0.36–0.74)	<0.001
ΔCt	ΔCt + Age	0.91 (0.89–0.95)	<0.001
Age 40–49 years	ΔCt + Age	0.84 (0.60–1.17)	0.293
Age 50–64 years	ΔCt + Age	0.55 (0.38–0.79)	0.001

Note: ORs for ΔCt represent the change in the odds of a high-grade histological lesion associated with a one-unit increase in ΔCt. Therefore, an OR below 1 indicates that higher ΔCt values are associated with lower odds of a high-grade lesion, or equivalently, that lower ΔCt values are associated with higher odds. Age was categorized as 30–39, 40–49, and 50–64 years, with women aged 30–39 years used as the reference group. The unadjusted model included ΔCt only, the age-only model included age category only, and the adjusted model included both ΔCt and age category.

**Table 4:** Age-adjusted detection-channel-stratified logistic regression analyses evaluating the association between ΔCt and high-grade histological lesions.

HPV Detection Channel	*n*. Analyzed	Missing	Predictor	OR	95% CI	*p*-Value
HPV16	200	0	ΔCt	0.87	0.816–0.923	<0.001
			Age 40–49 years	1.28	0.62–2.64	0.503
			Age 50–64 years	0.52	0.25–1.10	0.089
HPV18	58	0	ΔCt	0.93	0.84–1.03	0.167
			Age 40–49 years	3.28	0.55–19.64	0.193
			Age 50–64 years	4.48	0.73–27.38	0.104
Pooled 12-high-risk-HPV	788	0	ΔCt	0.93	0.897–0.954	<0.001
			Age 40–49 years	0.71	0.48–1.05	0.083
			Age 50–64 years	0.48	0.31–0.75	0.001

Note: Women aged 30–39 years were the reference age group.

## Data Availability

The dataset analyzed during the current study is not publicly available due to privacy and ethical restrictions associated with clinical screening data but is available from the corresponding author on reasonable request and with permission of the relevant ethics committee.

## Supplementary Materials

The following supporting information can be downloaded at: https://www.mdpi.com/article/10.3390/cancers18162568/s1, Table S1: Baseline characteristics of HPV-positive women according to histological verification status; Table S2: Detection Channel-specific differences in the relationship between RVL and disease progression; Table S3: Diagnostic performance of ΔCt for identifying high-grade cervical lesions using the ROC-derived threshold corresponding to the maximum Youden index; Table S4: Logistic regression analysis of the association between ΔCt and cytological lesion grade, with and without adjustment for age category.

## Abbreviations

The following abbreviations are used in this manuscript:

AC	Adenocarcinoma
AGCs	Atypical Glandular Cells
ASC-H	Atypical Squamous Cells—cannot exclude H-SIL
ASC-US	Atypical Squamous Cells of Undetermined Significance
AUC	Area Under the Curve
CIN1	Cervical Intraepithelial Neoplasia grade 1
CIN2	Cervical Intraepithelial Neoplasia grade 2
CIN3	Cervical Intraepithelial Neoplasia grade 3
Ct	Cycle threshold
HR	High Risk
HPV	Human Papillomavirus
H-SIL	High-Grade Squamous Intraepithelial Lesion
IQR	Interquartile Range
LBC	Liquid Based Cytology
LoD	Limit of Detection
L-SIL	Low-Grade Squamous Intraepithelial Lesion
NILM	Negative for Intraepithelial Lesion or Malignancy
OR	Odds Ratio
PCR	Polymerase Chain Reaction
ROC	Receiver Operating Characteristic
RVL	Relative Viral Load
SCC	Squamous Cell Carcinoma
