# Supplementary material for: Relative HPV Viral Load Measured by ΔCt for Risk Stratification in Cervical Cancer Screening
Source: Cancers (Basel). 2026 Aug 10;18(16):2568. doi: 10.3390/cancers18162568 (PMC13511412; doi:10.3390/cancers18162568)
Supplement: Supplementary file 1 [file cancers-18-02568-s001.zip › cancers-4408848-supplementary.pdf]

Supplementary Results

Supplementary Table S1. Baseline characteristics of HPV-positive women according to histological verification status.

Characteristic	*No histology (n = 1764)*	*Histology available (n = 997)*	*p-value*
**Age, median (IQR)**	45 (37–53)	44 (36–52)	0.085
**β-globin Ct, median (IQR)**	27.8 (27.0–28.8)	27.6 (26.8–28.4)	<0.001
**ΔCt, median (IQR)**	4.9 (0.3–9.0)	2.8 (−1.4–7.3)	<0.001
**Cytology**			<0.001
**• NILM**	837 (47.4%)	138 (13.8%)	
**• ASC-US / LSIL**	824 (46.7%)	609 (61.1%)	
**• ASC-H / HSIL**	58 (3.3%)	203 (20.4%)	
**• AGC / Adenocarcinoma**	6 (0.3%)	16 (1.6%)	
**• Inadequate**	39 (2.2%)	31 (3.1%)	
**HPV16 positive**	252 (14.3%)	214 (21.5%)	<0.001
**HPV18 positive**	108 (6.1%)	103 (10.3%)	<0.001
**Pooled 12-HR-HPVchannel positive**	1579 (89.5%)	851 (85.4%)	0.001

Footnote: Continuous variables are presented as median (interquartile range) and compared using the Mann–Whitney U test. Categorical variables are presented as number (%) and compared using Pearson's χ^2 test. Cytological categories were grouped for descriptive purposes only. Percentages for HPV16, HPV18 and Other HR HPV are not mutually exclusive because women with multiple HPV infections contributed to more than one genotype category.

Cytological Findings

A similar analysis was performed using cytological lesion grade as the outcome.

Consistent with the histological findings, a significant association between RVL and lesion severity was observed for HPV16 and other high-risk HPV genotypes, but not for HPV18.

Among HPV16-positive women, those with non-high-grade cytological lesions had a median ΔCt of 4.2 (IQR: 0.3–8.15), whereas those with high-grade cytological lesions had a

significantly lower median ΔCt of −0.5 (IQR: −2.7 to 3.0) ($p = 0.0001$), confirming that higher RVL was associated with increased cytological severity in HPV16 infections.

In contrast, HPV18 infections showed no statistically significant difference in RVL between lesion grades. The median ΔCt was 3.8 (IQR: −0.4 to 8.1) in non-high-grade lesions and 0.9 (IQR: −3.9 to 7.5) in high-grade lesions ($p = 0.9283$), indicating no clear association between RVL and cytological lesion severity for this genotype.

For other high-risk HPV genotypes, RVL was again significantly associated with lesion severity. Women with non-high-grade cytological lesions had a median ΔCt of 3.2 (IQR: −1.0 to 8.0), compared with a significantly lower median ΔCt of 0.6 (IQR: −3.2 to 4.6) in those with high-grade lesions ($p = 0.0002$). These findings further support the association between higher RVL and increased lesion severity among women infected with non-16/18 high-risk HPV genotypes.

Taken together, these results demonstrate a consistent pattern in which higher RVL is significantly associated with high-grade lesions for HPV16 and other high-risk HPV genotypes across both histological and cytological classifications. In contrast, HPV18 infections did not show a significant association between viral load and lesion severity in either analysis. These findings suggest genotype-specific differences in the relationship between RVL and disease progression, with HPV16 showing the strongest association with lesion severity as expected (Supplementary Table S2).

Supplementary Table S2. Detection Channel-specific differences in the relationship between RVL and disease progression

HPV type	Cytology grade lesion	*n*° of subject	ΔCt mean and ds	p-value
HPV 16	Non-high-grade	116	4.2 (0.3–8.15)	R
	High-grade	79	−0.5 (−2.7–3.0)	0.0001
HPV 18	Non-High-grade	37	3.8 (−0.4–8.1)	R
	High-grade	19	0.9 (−3.9–7.5)	0.9283
Pooled 12-HR-HPV detection channel	Non-high-grade	623	3.2 (−1.0–8.0)	R
	High-grade	138	0.6 (−3.2–4.6)	0.0002

Supplementary Table S3. Diagnostic performance of ΔCt for identifying high-grade cervical lesions using the ROC-derived threshold corresponding to the maximum Youden index.

Parameter	***Value***
**ROC AUC (95% CI)**	0.643 (0.605–0.681)
**Optimal ROC-derived threshold (maximum Youden index)**	**ΔCt = 1.0**
**Sensitivity**	55.9%
**Specificity**	67.6%
**Positive Predictive Value (PPV)**	39.8%
**Negative Predictive Value (NPV)**	80.0%

Association between cytology grade of lesions and RVL and age

Logistic regression analyses were conducted to examine the association between cytological lesion grade (non-high-grade- vs high-grade) and viral load, measured as ΔCt, both unadjusted and adjusted for age category. In the univariate model including ΔCt only, lower ΔCt values, indicative of higher viral load, were significantly associated with higher odds of high-grade cytological lesions, with each unit decrease in ΔCt corresponding to approximately a 6% increase in the likelihood of a high-grade lesion (OR = 0.94, 95% CI 0.91–0.96, $p < 0.001$), indicating a clear inverse relationship between ΔCt and lesion severity.

When age category was considered alone, no significant associations with cytological grade were observed; neither the intermediate age group (40–49 years ; OR = 0.93, 95% CI 0.65–1.34, p = 0.71) nor the oldest group (≥50 years; OR = 0.89, 95% CI 0.61–1.29, p = 0.53) showed statistically meaningful differences compared with the youngest category, suggesting that age does not strongly influence cytological lesion grade. In the multivariate model including both ΔCt and age category, the inverse association between ΔCt and high-grade lesions persisted (OR = 0.94, 95% CI 0.91–0.96, p < 0.001), confirming that RVL is independently associated with cytological lesion severity, while age effects remained non-significant for both intermediate (OR = 1.11, 95% CI 0.74–1.66, p = 0.59) and oldest age groups (OR = 0.91, 95% CI 0.59–1.41, p = 0.68). Overall, these findings indicate that ΔCt is a robust predictor of high-grade cytological lesions, whereas age does not appear to contribute meaningfully in this context (supplementary Table S4).

Supplementary Table S4. Logistic regression analysis of the association between ΔCt and cytological lesion grade, with and without adjustment for age category.

Model	Predictor	OR	95% CI	p-value
Unadjusted (ΔCt only)	ΔCt	0.94	0.91–0.96	<0.001
Age only	40–49 years	0.93	0.65–1.34	0.71
	≥50 years	0.89	0.61–1.29	0.53
Adjusted (ΔCt + age)	ΔCt	0.94	0.91–0.96	<0.001
	40–49 years	1.11	0.74–1.66	0.592
	≥50 years	0.91	0.58–1.41	0.682
